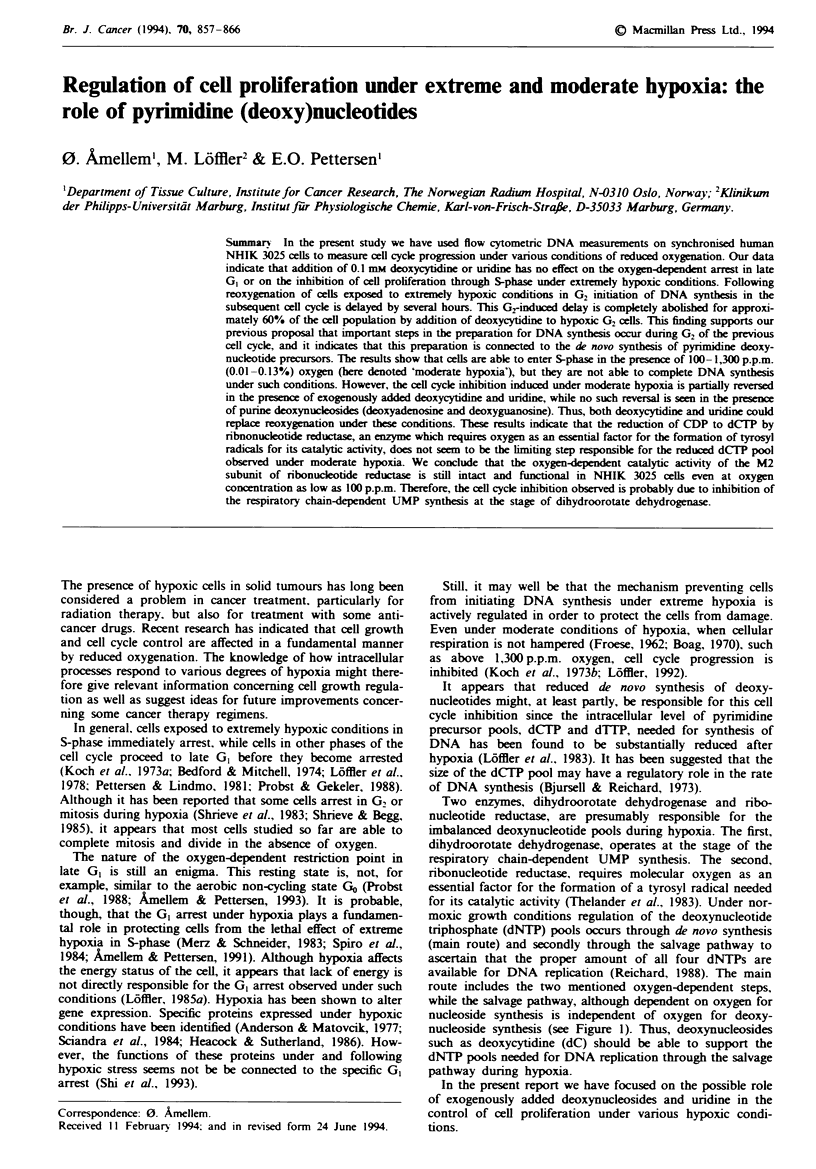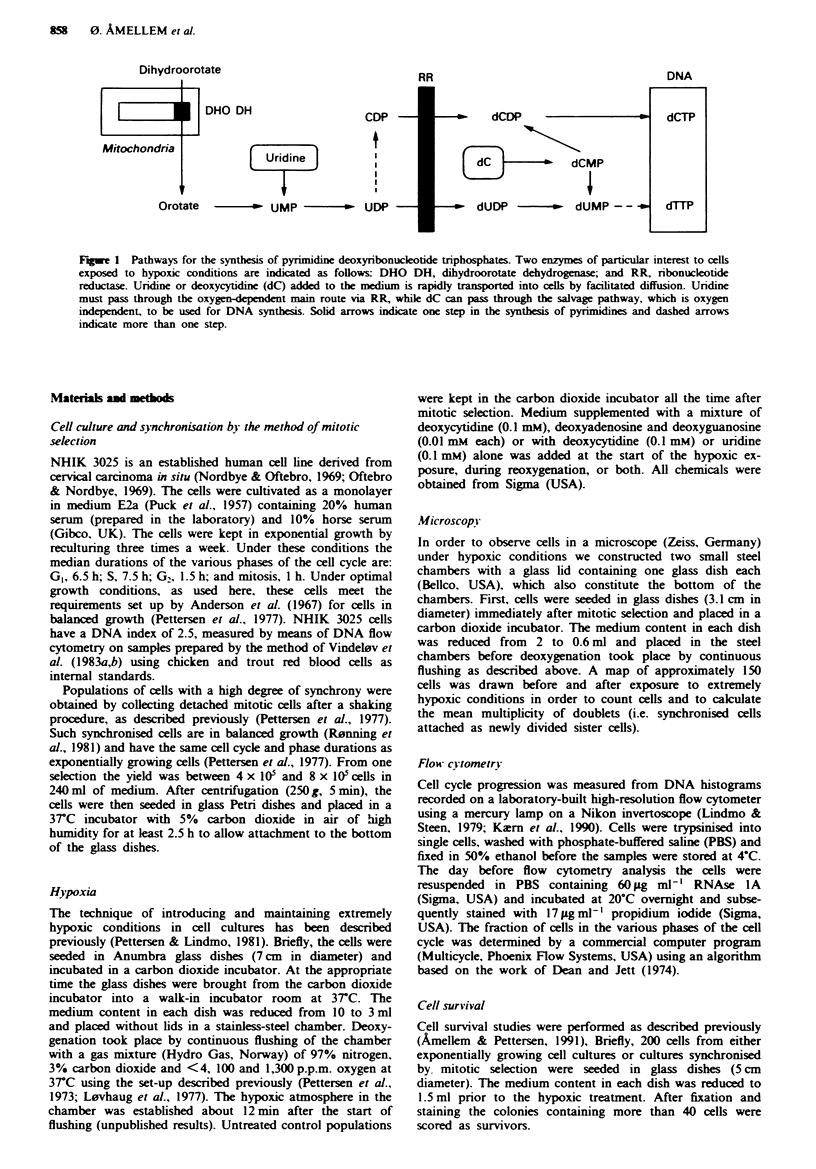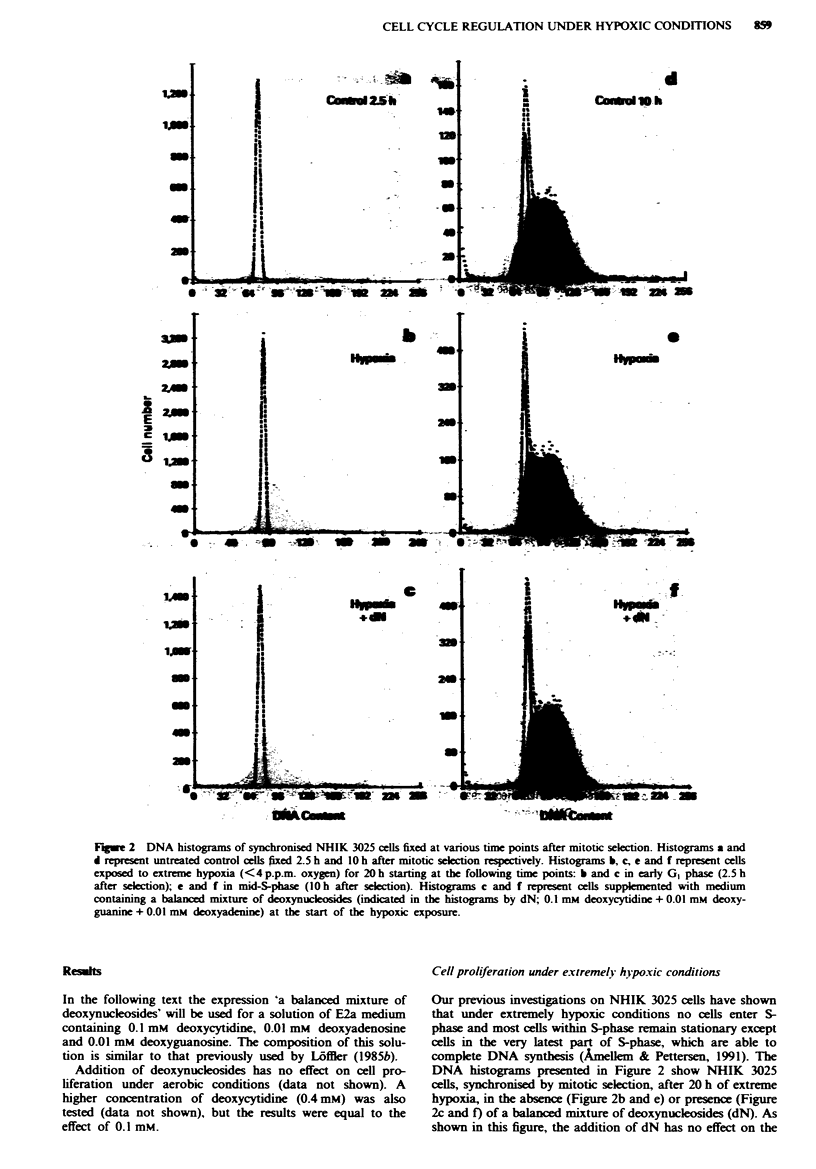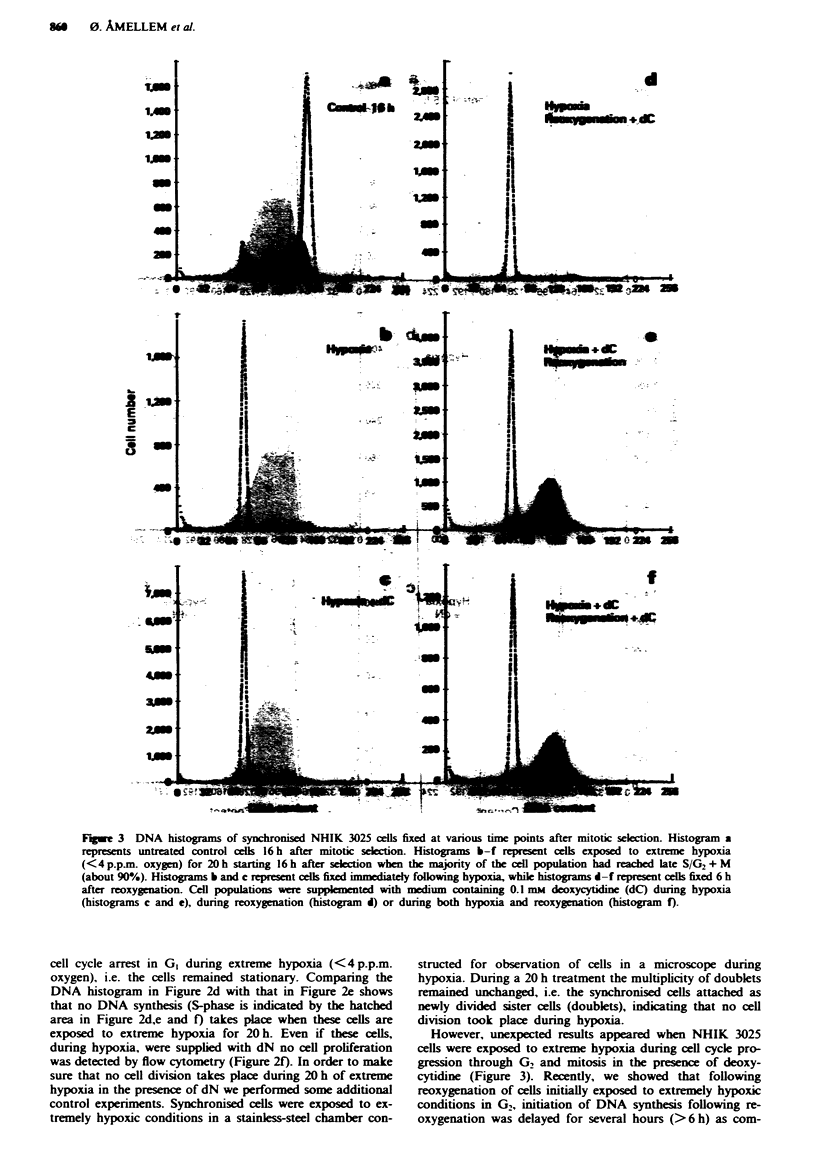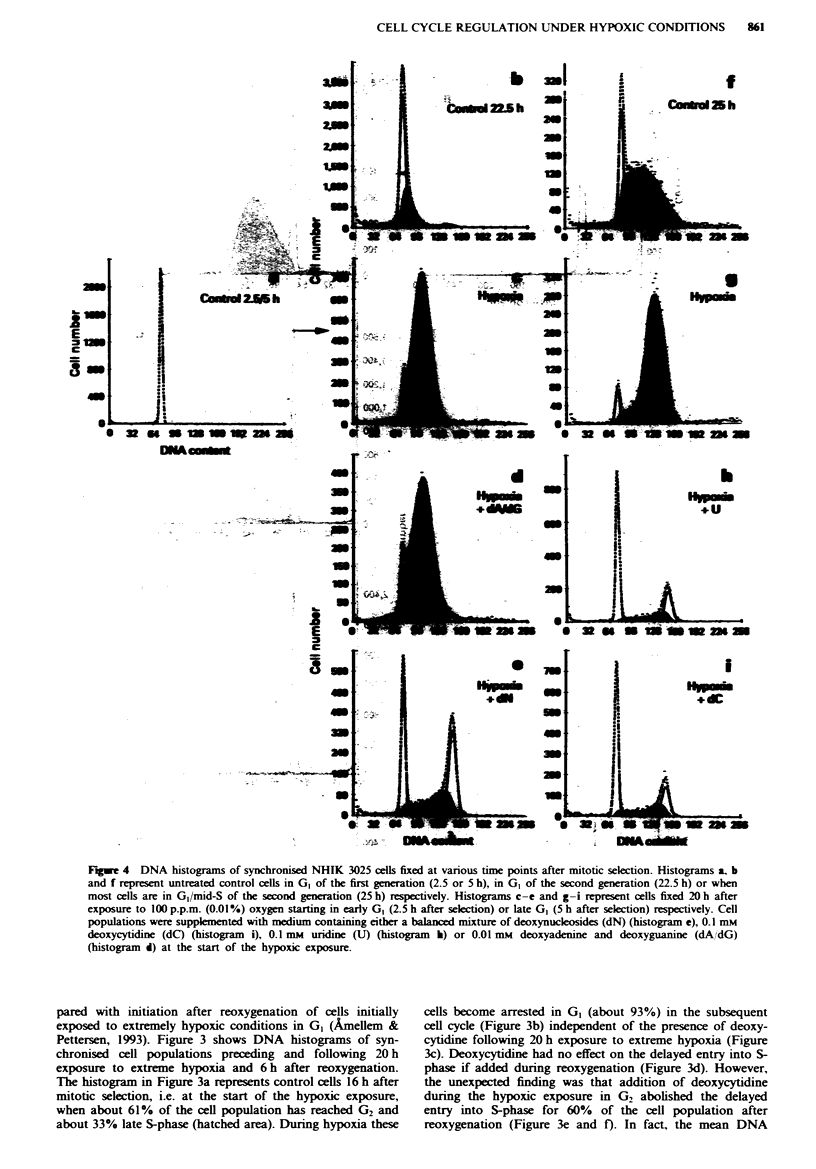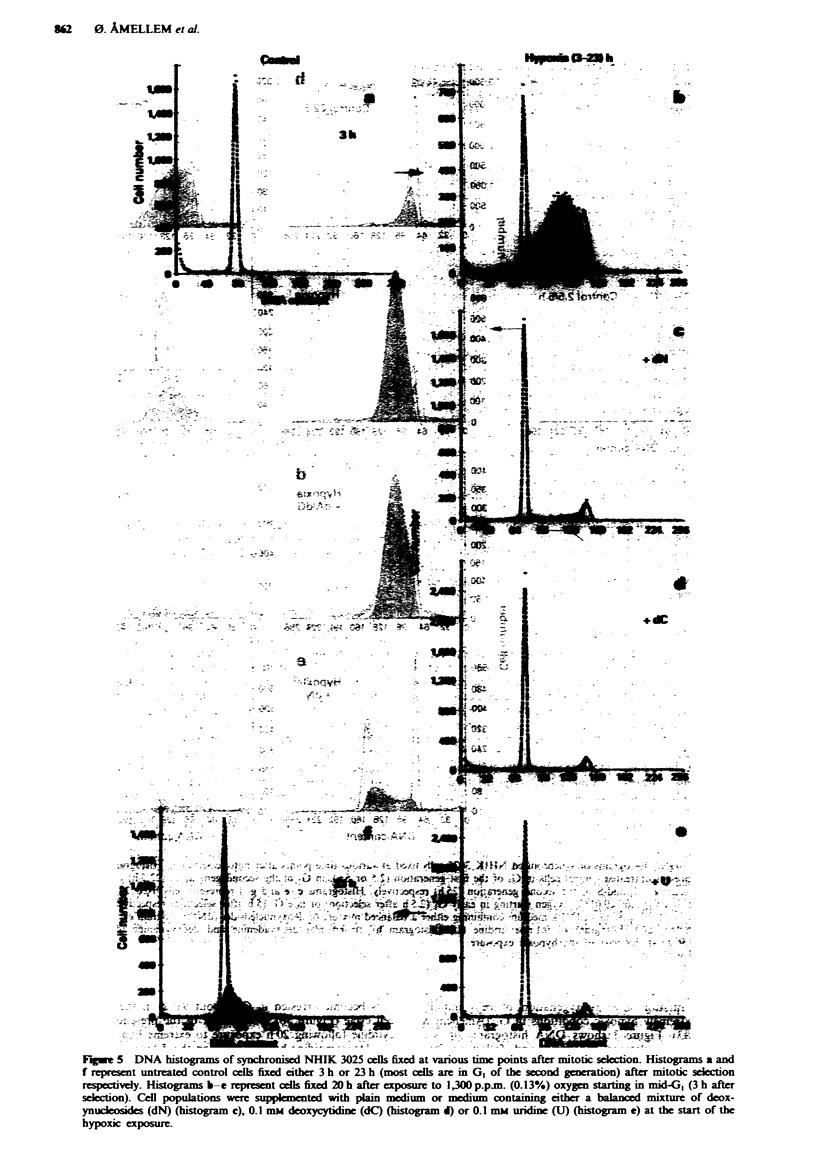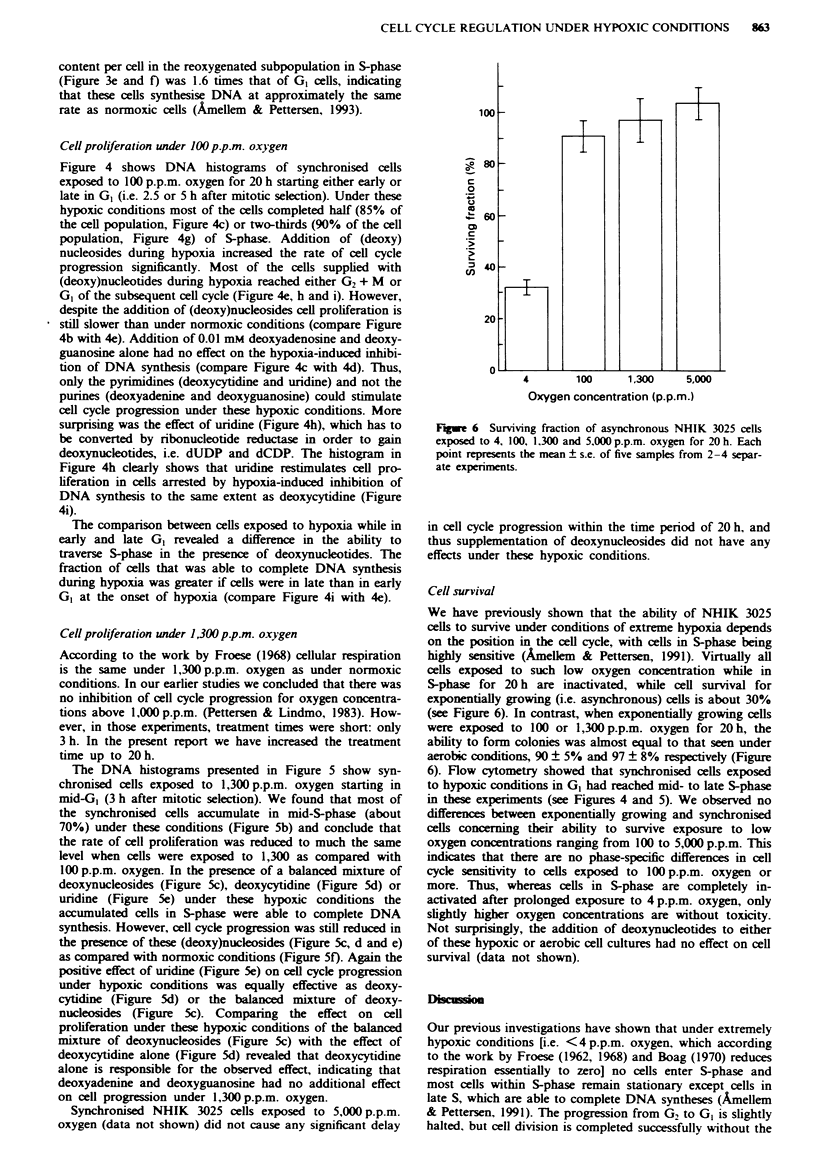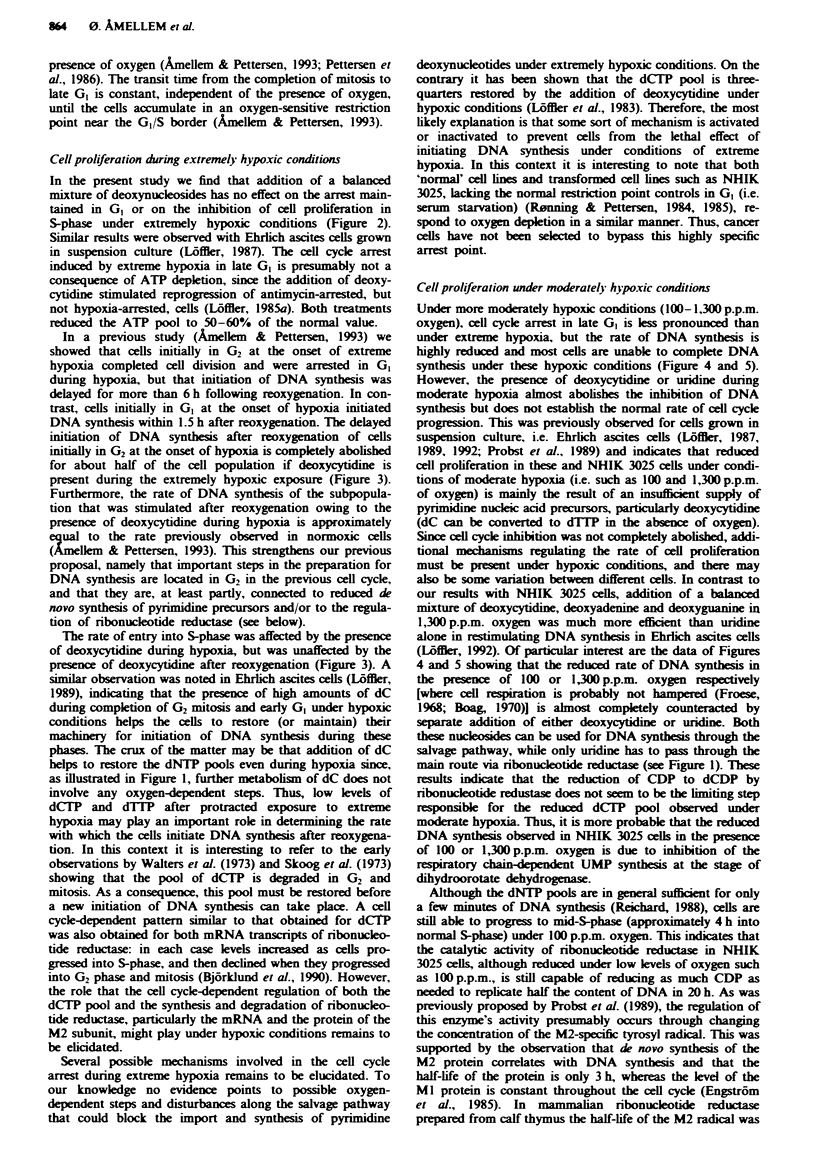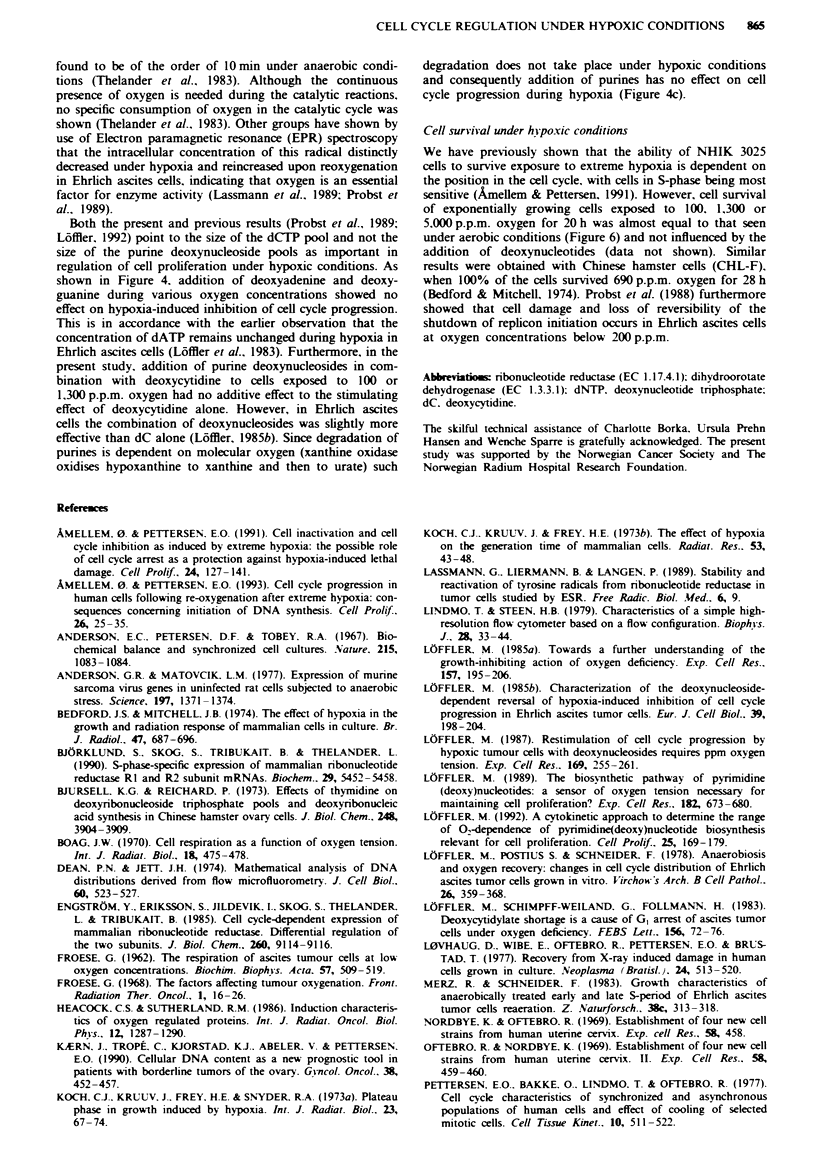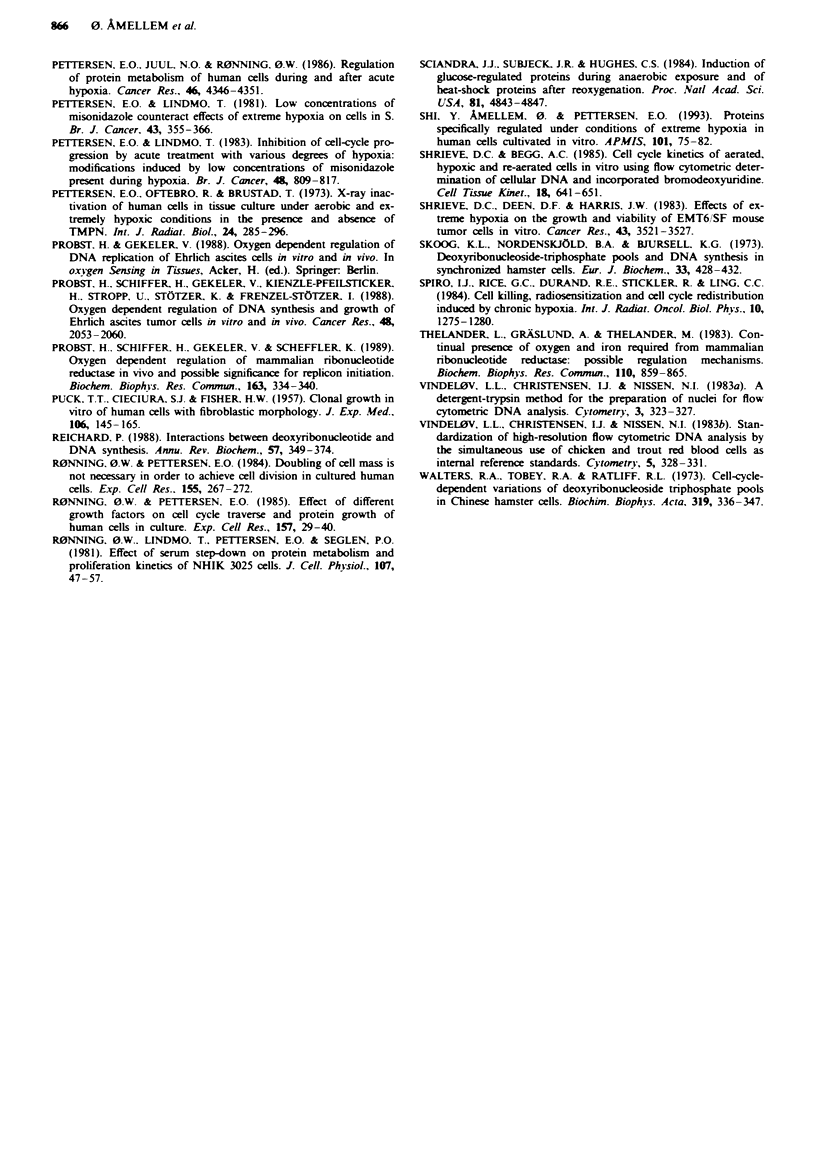# Regulation of cell proliferation under extreme and moderate hypoxia: the role of pyrimidine (deoxy)nucleotides.

**DOI:** 10.1038/bjc.1994.411

**Published:** 1994-11

**Authors:** O. Amellem, M. Löffler, E. O. Pettersen

**Affiliations:** Department of Tissue Culture, Norwegian Radium Hospital, Oslo.

## Abstract

In the present study we have used flow cytometric DNA measurements on synchronised human NHIK 3025 cells to measure cell cycle progression under various conditions of reduced oxygenation. Our data indicate that addition of 0.1 mM deoxycytidine or uridine has no effect on the oxygen-dependent arrest in late G1 or on the inhibition of cell proliferation through S-phase under extremely hypoxic conditions. Following reoxygenation of cells exposed to extremely hypoxic conditions in G2 initiation of DNA synthesis in the subsequent cell cycle is delayed by several hours. This G2-induced delay is completely abolished for approximately 60% of the cell population by addition of deoxycytidine to hypoxic G2 cells. This finding supports our previous proposal that important steps in the preparation for DNA synthesis occur during G2 of the previous cell cycle, and it indicates that this preparation is connected to the de novo synthesis of pyrimidine deoxynucleotide precursors. The results show that cells are able to enter S-phase in the presence of 100-1,300 p.p.m. (0.01-0.13%) oxygen (here denoted 'moderate hypoxia'), but they are not able to complete DNA synthesis under such conditions. However, the cell cycle inhibition induced under moderate hypoxia is partially reversed in the presence of exogenously added deoxycytidine and uridine, while no such reversal is seen in the presence of purine deoxynucleosides (deoxyadenosine and deoxyguanosine). Thus, both deoxycytidine and uridine could replace reoxygenation under these conditions. These results indicate that the reduction of CDP to dCTP by ribonucleotide reductase, an enzyme which requires oxygen as an essential factor for the formation of tyrosyl radicals for its catalytic activity, does not seem to be the limiting step responsible for the reduced dCTP pool observed under moderate hypoxia. We conclude that the oxygen-dependent catalytic activity of the M2 subunit of ribonucleotide reductase is still intact and functional in NHIK 3025 cells even at oxygen concentration as low as 100 p.p.m. Therefore the cell cycle inhibition observed is probably due to inhibition of the respiratory chain-dependent UMP synthesis at the stage of dihydroorotate dehydrogenase.


Br. J. Cancer (1994), 70, 857-866
© Macmillan Press Ltd., 1994

### Regulation of cell proliferation under extreme and moderate hypoxia: the role of pyrimidine (deoxy)nucleotides

Ø. Åmellem[1], M. Löffler[2] & E.O. Pettersen[1]

[1]Department of Tissue Culture, Institute for Cancer Research, The Norwegian Radium Hospital, N-0310 Oslo, Norway; [2]Klinikum der Philipps-Universität Marburg, Institut für Physiologische Chemie, Karl-von-Frisch-Straße, D-35033 Marburg, Germany.

**Summary** In the present study we have used flow cytometric DNA measurements on synchronised human NHIK 3025 cells to measure cell cycle progression under various conditions of reduced oxygenation. Our data indicate that addition of 0.1 mM deoxycytidine or uridine has no effect on the oxygen-dependent arrest in late $G_1$ or on the inhibition of cell proliferation through S-phase under extremely hypoxic conditions. Following reoxygenation of cells exposed to extremely hypoxic conditions in $G_2$ initiation of DNA synthesis in the subsequent cell cycle is delayed by several hours. This $G_2$-induced delay is completely abolished for approximately 60% of the cell population by addition of deoxycytidine to hypoxic $G_2$ cells. This finding supports our previous proposal that important steps in the preparation for DNA synthesis occur during $G_2$ of the previous cell cycle, and it indicates that this preparation is connected to the *de novo* synthesis of pyrimidine deoxynucleotide precursors. The results show that cells are able to enter S-phase in the presence of 100-1,300 p.p.m. (0.01-0.13%) oxygen (here denoted 'moderate hypoxia'), but they are not able to complete DNA synthesis under such conditions. However, the cell cycle inhibition induced under moderate hypoxia is partially reversed in the presence of exogenously added deoxycytidine and uridine, while no such reversal is seen in the presence of purine deoxynucleosides (deoxyadenosine and deoxyguanosine). Thus, both deoxycytidine and uridine could replace reoxygenation under these conditions. These results indicate that the reduction of CDP to dCTP by ribnonucleotide reductase, an enzyme which requires oxygen as an essential factor for the formation of tyrosyl radicals for its catalytic activity, does not seem to be the limiting step responsible for the reduced dCTP pool observed under moderate hypoxia. We conclude that the oxygen-dependent catalytic activity of the M2 subunit of ribonucleotide reductase is still intact and functional in NHIK 3025 cells even at oxygen concentration as low as 100 p.p.m. Therefore, the cell cycle inhibition observed is probably due to inhibition of the respiratory chain-dependent UMP synthesis at the stage of dihydroorotate dehydrogenase.

The presence of hypoxic cells in solid tumours has long been considered a problem in cancer treatment, particularly for radiation therapy, but also for treatment with some anticancer drugs. Recent research has indicated that cell growth and cell cycle control are affected in a fundamental manner by reduced oxygenation. The knowledge of how intracellular processes respond to various degrees of hypoxia might therefore give relevant information concerning cell growth regulation as well as suggest ideas for future improvements concerning some cancer therapy regimens.

In general, cells exposed to extremely hypoxic conditions in S-phase immediately arrest, while cells in other phases of the cell cycle proceed to late $G_1$ before they become arrested (Koch *et al.*, 1973*a*; Bedford & Mitchell, 1974; Löffler *et al.*, 1978; Pettersen & Lindmo, 1981; Probst & Gekeler, 1988). Although it has been reported that some cells arrest in $G_2$ or mitosis during hypoxia (Shrieve *et al.*, 1983; Shrieve & Begg, 1985), it appears that most cells studied so far are able to complete mitosis and divide in the absence of oxygen.

The nature of the oxygen-dependent restriction point in late $G_1$ is still an enigma. This resting state is, not, for example, similar to the aerobic non-cycling state $G_0$ (Probst *et al.*, 1988; Åmellem & Pettersen, 1993). It is probable, though, that the $G_1$ arrest under hypoxia plays a fundamental role in protecting cells from the lethal effect of extreme hypoxia in S-phase (Merz & Schneider, 1983; Spiro *et al.*, 1984; Åmellem & Pettersen, 1991). Although hypoxia affects the energy status of the cell, it appears that lack of energy is not directly responsible for the $G_1$ arrest observed under such conditions (Löffler, 1985*a*). Hypoxia has been shown to alter gene expression. Specific proteins expressed under hypoxic conditions have been identified (Anderson & Matovcik, 1977; Sciandra *et al.*, 1984; Heacock & Sutherland, 1986). However, the functions of these proteins under and following hypoxic stress seems not be be connected to the specific $G_1$ arrest (Shi *et al.*, 1993).

Correspondence: Ø. Åmellem.

Received 11 February 1994; and in revised form 24 June 1994.

Still, it may well be that the mechanism preventing cells from initiating DNA synthesis under extreme hypoxia is actively regulated in order to protect the cells from damage. Even under moderate conditions of hypoxia, when cellular respiration is not hampered (Froese, 1962; Boag, 1970), such as above 1,300 p.p.m. oxygen, cell cycle progression is inhibited (Koch *et al.*, 1973*b*; Löffler, 1992).

It appears that reduced *de novo* synthesis of deoxynucleotides might, at least partly, be responsible for this cell cycle inhibition since the intracellular level of pyrimidine precursor pools, dCTP and dTTP, needed for synthesis of DNA has been found to be substantially reduced after hypoxia (Löffler *et al.*, 1983). It has been suggested that the size of the dCTP pool may have a regulatory role in the rate of DNA synthesis (Bjursell & Reichard, 1973).

Two enzymes, dihydroorotate dehydrogenase and ribonucleotide reductase, are presumably responsible for the imbalanced deoxynucleotide pools during hypoxia. The first, dihydroorotate dehydrogenase, operates at the stage of the respiratory chain-dependent UMP synthesis. The second, ribonucleotide reductase, requires molecular oxygen as an essential factor for the formation of a tyrosyl radical needed for its catalytic activity (Thelander *et al.*, 1983). Under normoxic growth conditions regulation of the deoxynucleotide triphosphate (dNTP) pools occurs through *de novo* synthesis (main route) and secondly through the salvage pathway to ascertain that the proper amount of all four dNTPs are available for DNA replication (Reichard, 1988). The main route includes the two mentioned oxygen-dependent steps, while the salvage pathway, although dependent on oxygen for nucleoside synthesis is independent of oxygen for deoxynucleoside synthesis (see Figure 1). Thus, deoxynucleosides such as deoxycytidine (dC) should be able to support the dNTP pools needed for DNA replication through the salvage pathway during hypoxia.

In the present report we have focused on the possible role of exogenously added deoxynucleosides and uridine in the control of cell proliferation under various hypoxic conditions.

**Figure 1** Pathways for the synthesis of pyrimidine deoxyribonucleotide triphosphates. Two enzymes of particular interest to cells exposed to hypoxic conditions are indicated as follows: DHO DH, dihydroorotate dehydrogenase; and RR, ribonucleotide reductase. Uridine or deoxycytidine (dC) added to the medium is rapidly transported into cells by facilitated diffusion. Uridine must pass through the oxygen-dependent main route via RR, while dC can pass through the salvage pathway, which is oxygen independent, to be used for DNA synthesis. Solid arrows indicate one step in the synthesis of pyrimidines and dashed arrows indicate more than one step.

### Materials and methods

### *Cell culture and synchronisation by the method of mitotic selection*

NHIK 3025 is an established human cell line derived from cervical carcinoma *in situ* (Nordbye & Oftebro, 1969; Oftebro & Nordbye, 1969). The cells were cultivated as a monolayer in medium E2a (Puck *et al.*, 1957) containing 20% human serum (prepared in the laboratory) and 10% horse serum (Gibco, UK). The cells were kept in exponential growth by reculturing three times a week. Under these conditions the median durations of the various phases of the cell cycle are: $G_1$, 6.5 h; S, 7.5 h; $G_2$, 1.5 h; and mitosis, 1 h. Under optimal growth conditions, as used here, these cells meet the requirements set up by Anderson *et al.* (1967) for cells in balanced growth (Pettersen *et al.*, 1977). NHIK 3025 cells have a DNA index of 2.5, measured by means of DNA flow cytometry on samples prepared by the method of Vindeløv *et al.* (1983*a*,*b*) using chicken and trout red blood cells as internal standards.

Populations of cells with a high degree of synchrony were obtained by collecting detached mitotic cells after a shaking procedure, as described previously (Pettersen *et al.*, 1977). Such synchronised cells are in balanced growth (Rønning *et al.*, 1981) and have the same cell cycle and phase durations as exponentially growing cells (Pettersen *et al.*, 1977). From one selection the yield was between $4 \times 10^5$ and $8 \times 10^5$ cells in 240 ml of medium. After centrifugation (250 *g*, 5 min), the cells were then seeded in glass Petri dishes and placed in a 37°C incubator with 5% carbon dioxide in air of high humidity for at least 2.5 h to allow attachment to the bottom of the glass dishes.

### *Hypoxia*

The technique of introducing and maintaining extremely hypoxic conditions in cell cultures has been described previously (Pettersen & Lindmo, 1981). Briefly, the cells were seeded in Anumbra glass dishes (7 cm in diameter) and incubated in a carbon dioxide incubator. At the appropriate time the glass dishes were brought from the carbon dioxide incubator into a walk-in incubator room at 37°C. The medium content in each dish was reduced from 10 to 3 ml and placed without lids in a stainless-steel chamber. Deoxygenation took place by continuous flushing of the chamber with a gas mixture (Hydro Gas, Norway) of 97% nitrogen, 3% carbon dioxide and $<4$, 100 and 1,300 p.p.m. oxygen at 37°C using the set-up described previously (Pettersen *et al.*, 1973; Løvhaug *et al.*, 1977). The hypoxic atmosphere in the chamber was established about 12 min after the start of flushing (unpublished results). Untreated control populations were kept in the carbon dioxide incubator all the time after mitotic selection. Medium supplemented with a mixture of deoxycytidine (0.1 mM), deoxyadenosine and deoxyguanosine (0.01 mM each) or with deoxycytidine (0.1 mM) or uridine (0.1 mM) alone was added at the start of the hypoxic exposure, during reoxygenation, or both. All chemicals were obtained from Sigma (USA).

### *Microscopy*

In order to observe cells in a microscope (Zeiss, Germany) under hypoxic conditions we constructed two small steel chambers with a glass lid containing one glass dish each (Bellco, USA), which also constitute the bottom of the chambers. First, cells were seeded in glass dishes (3.1 cm in diameter) immediately after mitotic selection and placed in a carbon dioxide incubator. The medium content in each dish was reduced from 2 to 0.6 ml and placed in the steel chambers before deoxygenation took place by continuous flushing as described above. A map of approximately 150 cells was drawn before and after exposure to extremely hypoxic conditions in order to count cells and to calculate the mean multiplicity of doublets (i.e. synchronised cells attached as newly divided sister cells).

### *Flow cytometry*

Cell cycle progression was measured from DNA histograms recorded on a laboratory-built high-resolution flow cytometer using a mercury lamp on a Nikon invertoscope (Lindmo & Steen, 1979; Kærn *et al.*, 1990). Cells were trypsinised into single cells, washed with phosphate-buffered saline (PBS) and fixed in 50% ethanol before the samples were stored at 4°C. The day before flow cytometry analysis the cells were resuspended in PBS containing 60 μg $ml^{-1}$ RNAse 1A (Sigma, USA) and incubated at 20°C overnight and subsequently stained with 17 μg $ml^{-1}$ propidium iodide (Sigma, USA). The fraction of cells in the various phases of the cell cycle was determined by a commercial computer program (Multicycle, Phoenix Flow Systems, USA) using an algorithm based on the work of Dean and Jett (1974).

### *Cell survival*

Cell survival studies were performed as described previously (Åmellem & Pettersen, 1991). Briefly, 200 cells from either exponentially growing cell cultures or cultures synchronised by mitotic selection were seeded in glass dishes (5 cm diameter). The medium content in each dish was reduced to 1.5 ml prior to the hypoxic treatment. After fixation and staining the colonies containing more than 40 cells were scored as survivors.

**Figure 2** DNA histograms of synchronised NHIK 3025 cells fixed at various time points after mitotic selection. Histograms **a** and **d** represent untreated control cells fixed 2.5 h and 10 h after mitotic selection respectively. Histograms **b**, **c**, **e** and **f** represent cells exposed to extreme hypoxia (<4 p.p.m. oxygen) for 20 h starting at the following time points: **b** and **c** in early $G_1$ phase (2.5 h after selection); **e** and **f** in mid-S-phase (10 h after selection). Histograms **c** and **f** represent cells supplemented with medium containing a balanced mixture of deoxynucleosides (indicated in the histograms by dN; 0.1 mM deoxycytidine + 0.01 mM deoxyguanine + 0.01 mM deoxyadenine) at the start of the hypoxic exposure.

### Results

In the following text the expression 'a balanced mixture of deoxynucleosides' will be used for a solution of E2a medium containing 0.1 mM deoxycytidine, 0.01 mM deoxyadenosine and 0.01 mM deoxyguanosine. The composition of this solution is similar to that previously used by Löffler (1985*b*).

Addition of deoxynucleosides has no effect on cell proliferation under aerobic conditions (data not shown). A higher concentration of deoxycytidine (0.4 mM) was also tested (data not shown), but the results were equal to the effect of 0.1 mM.

### *Cell proliferation under extremely hypoxic conditions*

Our previous investigations on NHIK 3025 cells have shown that under extremely hypoxic conditions no cells enter S-phase and most cells within S-phase remain stationary except cells in the very latest part of S-phase, which are able to complete DNA synthesis (Åmellem & Pettersen, 1991). The DNA histograms presented in Figure 2 show NHIK 3025 cells, synchronised by mitotic selection, after 20 h of extreme hypoxia, in the absence (Figure 2b and e) or presence (Figure 2c and f) of a balanced mixture of deoxynucleosides (dN). As shown in this figure, the addition of dN has no effect on the

**Figure 3** DNA histograms of synchronised NHIK 3025 cells fixed at various time points after mitotic selection. Histogram **a** represents untreated control cells 16 h after mitotic selection. Histograms **b**–**f** represent cells exposed to extreme hypoxia (<4 p.p.m. oxygen) for 20 h starting 16 h after selection when the majority of the cell population had reached late $S/G_2 + M$ (about 90%). Histograms **b** and **c** represent cells fixed immediately following hypoxia, while histograms **d**–**f** represent cells fixed 6 h after reoxygenation. Cell populations were supplemented with medium containing 0.1 mM deoxycytidine (dC) during hypoxia (histograms **c** and **e**), during reoxygenation (histogram **d**) or during both hypoxia and reoxygenation (histogram **f**).

cell cycle arrest in $G_1$ during extreme hypoxia (<4 p.p.m. oxygen), i.e. the cells remained stationary. Comparing the DNA histogram in Figure 2d with that in Figure 2e shows that no DNA synthesis (S-phase is indicated by the hatched area in Figure 2d,e and f) takes place when these cells are exposed to extreme hypoxia for 20 h. Even if these cells, during hypoxia, were supplied with dN no cell proliferation was detected by flow cytometry (Figure 2f). In order to make sure that no cell division takes place during 20 h of extreme hypoxia in the presence of dN we performed some additional control experiments. Synchronised cells were exposed to extremely hypoxic conditions in a stainless-steel chamber constructed for observation of cells in a microscope during hypoxia. During a 20 h treatment the multiplicity of doublets remained unchanged, i.e. the synchronised cells attached as newly divided sister cells (doublets), indicating that no cell division took place during hypoxia.

However, unexpected results appeared when NHIK 3025 cells were exposed to extreme hypoxia during cell cycle progression through $G_2$ and mitosis in the presence of deoxycytidine (Figure 3). Recently, we showed that following reoxygenation of cells initially exposed to extremely hypoxic conditions in $G_2$, initiation of DNA synthesis following reoxygenation was delayed for several hours (>6 h) as com-

**Figure 4** DNA histograms of synchronised NHIK 3025 cells fixed at various time points after mitotic selection. Histograms **a**, **b** and **f** represent untreated control cells in $G_1$ of the first generation (2.5 or 5 h), in $G_1$ of the second generation (22.5 h) or when most cells are in $G_1$/mid-S of the second generation (25 h) respectively. Histograms **c**–**e** and **g**–**i** represent cells fixed 20 h after exposure to 100 p.p.m. (0.01%) oxygen starting in early $G_1$ (2.5 h after selection) or late $G_1$ (5 h after selection) respectively. Cell populations were supplemented with medium containing either a balanced mixture of deoxynucleosides (dN) (histogram **e**), 0.1 mM deoxycytidine (dC) (histogram **i**), 0.1 mM uridine (U) (histogram **h**) or 0.01 mM deoxyadenine and deoxyguanine (dA/dG) (histogram **d**) at the start of the hypoxic exposure.

pared with initiation after reoxygenation of cells initially exposed to extremely hypoxic conditions in $G_1$ (Åmellem & Pettersen, 1993). Figure 3 shows DNA histograms of synchronised cell populations preceding and following 20 h exposure to extreme hypoxia and 6 h after reoxygenation. The histogram in Figure 3a represents control cells 16 h after mitotic selection, i.e. at the start of the hypoxic exposure, when about 61% of the cell population has reached $G_2$ and about 33% late S-phase (hatched area). During hypoxia these cells become arrested in $G_1$ (about 93%) in the subsequent cell cycle (Figure 3b) independent of the presence of deoxycytidine following 20 h exposure to extreme hypoxia (Figure 3c). Deoxycytidine had no effect on the delayed entry into S-phase if added during reoxygenation (Figure 3d). However, the unexpected finding was that addition of deoxycytidine during the hypoxic exposure in $G_2$ abolished the delayed entry into S-phase for 60% of the cell population after reoxygenation (Figure 3e and f). In fact, the mean DNA

**Figure 5** DNA histograms of synchronised NHIK 3025 cells fixed at various time points after mitotic selection. Histograms **a** and **f** represent untreated control cells fixed either 3 h or 23 h (most cells are in $G_1$ of the second generation) after mitotic selection respectively. Histograms **b**–**e** represent cells fixed 20 h after exposure to 1,300 p.p.m. (0.13%) oxygen starting in mid-$G_1$ (3 h after selection). Cell populations were supplemented with plain medium or medium containing either a balanced mixture of deoxynucleosides (dN) (histogram **c**), 0.1 mM deoxycytidine (dC) (histogram **d**) or 0.1 mM uridine (U) (histogram **e**) at the start of the hypoxic exposure.

content per cell in the reoxygenated subpopulation in S-phase (Figure 3e and f) was 1.6 times that of $G_1$ cells, indicating that these cells synthesise DNA at approximately the same rate as normoxic cells (Åmellem & Pettersen, 1993).

*Cell proliferation under 100 p.p.m. oxygen*

Figure 4 shows DNA histograms of synchronised cells exposed to 100 p.p.m. oxygen for 20 h starting either early or late in $G_1$ (i.e. 2.5 or 5 h after mitotic selection). Under these hypoxic conditions most of the cells completed half (85% of the cell population, Figure 4c) or two-thirds (90% of the cell population, Figure 4g) of S-phase. Addition of (deoxy) nucleosides during hypoxia increased the rate of cell cycle progression significantly. Most of the cells supplied with (deoxy)nucleotides during hypoxia reached either $G_2 + M$ or $G_1$ of the subsequent cell cycle (Figure 4e, h and i). However, despite the addition of (deoxy)nucleosides cell proliferation is still slower than under normoxic conditions (compare Figure 4b with 4e). Addition of 0.01 mM deoxyadenosine and deoxyguanosine alone had no effect on the hypoxia-induced inhibition of DNA synthesis (compare Figure 4c with 4d). Thus, only the pyrimidines (deoxycytidine and uridine) and not the purines (deoxyadenine and deoxyguanosine) could stimulate cell cycle progression under these hypoxic conditions. More surprising was the effect of uridine (Figure 4h), which has to be converted by ribonucleotide reductase in order to gain deoxynucleotides, i.e. dUDP and dCDP. The histogram in Figure 4h clearly shows that uridine restimulates cell proliferation in cells arrested by hypoxia-induced inhibition of DNA synthesis to the same extent as deoxycytidine (Figure 4i).

The comparison between cells exposed to hypoxia while in early and late $G_1$ revealed a difference in the ability to traverse S-phase in the presence of deoxynucleotides. The fraction of cells that was able to complete DNA synthesis during hypoxia was greater if cells were in late than in early $G_1$ at the onset of hypoxia (compare Figure 4i with 4e).

*Cell proliferation under 1,300 p.p.m. oxygen*

According to the work by Froese (1968) cellular respiration is the same under 1,300 p.p.m. oxygen as under normoxic conditions. In our earlier studies we concluded that there was no inhibition of cell cycle progression for oxygen concentrations above 1,000 p.p.m. (Pettersen & Lindmo, 1983). However, in those experiments, treatment times were short: only 3 h. In the present report we have increased the treatment time up to 20 h.

The DNA histograms presented in Figure 5 show synchronised cells exposed to 1,300 p.p.m. oxygen starting in mid-$G_1$ (3 h after mitotic selection). We found that most of the synchronised cells accumulate in mid-S-phase (about 70%) under these conditions (Figure 5b) and conclude that the rate of cell proliferation was reduced to much the same level when cells were exposed to 1,300 as compared with 100 p.p.m. oxygen. In the presence of a balanced mixture of deoxynucleosides (Figure 5c), deoxycytidine (Figure 5d) or uridine (Figure 5e) under these hypoxic conditions the accumulated cells in S-phase were able to complete DNA synthesis. However, cell cycle progression was still reduced in the presence of these (deoxy)nucleosides (Figure 5c, d and e) as compared with normoxic conditions (Figure 5f). Again the positive effect of uridine (Figure 5e) on cell cycle progression under hypoxic conditions was equally effective as deoxycytidine (Figure 5d) or the balanced mixture of deoxynucleosides (Figure 5c). Comparing the effect on cell proliferation under these hypoxic conditions of the balanced mixture of deoxynucleosides (Figure 5c) with the effect of deoxycytidine alone (Figure 5d) revealed that deoxycytidine alone is responsible for the observed effect, indicating that deoxyadenine and deoxyguanosine had no additional effect on cell progression under 1,300 p.p.m. oxygen.

Synchronised NHIK 3025 cells exposed to 5,000 p.p.m. oxygen (data not shown) did not cause any significant delay in cell cycle progression within the time period of 20 h, and thus supplementation of deoxynucleosides did not have any effects under these hypoxic conditions.

**Figure 6** Surviving fraction of asynchronous NHIK 3025 cells exposed to 4, 100, 1,300 and 5,000 p.p.m. oxygen for 20 h. Each point represents the mean ± s.e. of five samples from 2–4 separate experiments.

*Cell survival*

We have previously shown that the ability of NHIK 3025 cells to survive under conditions of extreme hypoxia depends on the position in the cell cycle, with cells in S-phase being highly sensitive (Åmellem & Pettersen, 1991). Virtually all cells exposed to such low oxygen concentration while in S-phase for 20 h are inactivated, while cell survival for exponentially growing (i.e. asynchronous) cells is about 30% (see Figure 6). In contrast, when exponentially growing cells were exposed to 100 or 1,300 p.p.m. oxygen for 20 h, the ability to form colonies was almost equal to that seen under aerobic conditions, 90 ± 5% and 97 ± 8% respectively (Figure 6). Flow cytometry showed that synchronised cells exposed to hypoxic conditions in $G_1$ had reached mid- to late S-phase in these experiments (see Figures 4 and 5). We observed no differences between exponentially growing and synchronised cells concerning their ability to survive exposure to low oxygen concentrations ranging from 100 to 5,000 p.p.m. This indicates that there are no phase-specific differences in cell cycle sensitivity to cells exposed to 100 p.p.m. oxygen or more. Thus, whereas cells in S-phase are completely inactivated after prolonged exposure to 4 p.p.m. oxygen, only slightly higher oxygen concentrations are without toxicity. Not surprisingly, the addition of deoxynucleotides to either of these hypoxic or aerobic cell cultures had no effect on cell survival (data not shown).

### Discussion

Our previous investigations have shown that under extremely hypoxic conditions [i.e. $<4$ p.p.m. oxygen, which according to the work by Froese (1962, 1968) and Boag (1970) reduces respiration essentially to zero] no cells enter S-phase and most cells within S-phase remain stationary except cells in late S, which are able to complete DNA syntheses (Åmellem & Pettersen, 1991). The progression from $G_2$ to $G_1$ is slightly halted, but cell division is completed successfully without the presence of oxygen (Åmellem & Pettersen, 1993; Pettersen et al., 1986). The transit time from the completion of mitosis to late $G_1$ is constant, independent of the presence of oxygen, until the cells accumulate in an oxygen-sensitive restriction point near the $G_1$/S border (Åmellem & Pettersen, 1993).

*Cell proliferation during extremely hypoxic conditions*

In the present study we find that addition of a balanced mixture of deoxynucleosides has no effect on the arrest maintained in $G_1$ or on the inhibition of cell proliferation in S-phase under extremely hypoxic conditions (Figure 2). Similar results were observed with Ehrlich ascites cells grown in suspension culture (Löffler, 1987). The cell cycle arrest induced by extreme hypoxia in late $G_1$ is presumably not a consequence of ATP depletion, since the addition of deoxycytidine stimulated reprogression of antimycin-arrested, but not hypoxia-arrested, cells (Löffler, 1985a). Both treatments reduced the ATP pool to 50–60% of the normal value.

In a previous study (Åmellem & Pettersen, 1993) we showed that cells initially in $G_2$ at the onset of extreme hypoxia completed cell division and were arrested in $G_1$ during hypoxia, but that initiation of DNA synthesis was delayed for more than 6 h following reoxygenation. In contrast, cells initially in $G_1$ at the onset of hypoxia initiated DNA synthesis within 1.5 h after reoxygenation. The delayed initiation of DNA synthesis after reoxygenation of cells initially in $G_2$ at the onset of hypoxia is completely abolished for about half of the cell population if deoxycytidine is present during the extremely hypoxic exposure (Figure 3). Furthermore, the rate of DNA synthesis of the subpopulation that was stimulated after reoxygenation owing to the presence of deoxycytidine during hypoxia is approximately equal to the rate previously observed in normoxic cells (Åmellem & Pettersen, 1993). This strengthens our previous proposal, namely that important steps in the preparation for DNA synthesis are located in $G_2$ in the previous cell cycle, and that they are, at least partly, connected to reduced *de novo* synthesis of pyrimidine precursors and/or to the regulation of ribonucleotide reductase (see below).

The rate of entry into S-phase was affected by the presence of deoxycytidine during hypoxia, but was unaffected by the presence of deoxycytidine after reoxygenation (Figure 3). A similar observation was noted in Ehrlich ascites cells (Löffler, 1989), indicating that the presence of high amounts of dC during completion of $G_2$ mitosis and early $G_1$ under hypoxic conditions helps the cells to restore (or maintain) their machinery for initiation of DNA synthesis during these phases. The crux of the matter may be that addition of dC helps to restore the dNTP pools even during hypoxia since, as illustrated in Figure 1, further metabolism of dC does not involve any oxygen-dependent steps. Thus, low levels of dCTP and dTTP after protracted exposure to extreme hypoxia may play an important role in determining the rate with which the cells initiate DNA synthesis after reoxygenation. In this context it is interesting to refer to the early observations by Walters et al. (1973) and Skoog et al. (1973) showing that the pool of dCTP is degraded in $G_2$ and mitosis. As a consequence, this pool must be restored before a new initiation of DNA synthesis can take place. A cell cycle-dependent pattern similar to that obtained for dCTP was also obtained for both mRNA transcripts of ribonucleotide reductase: in each case levels increased as cells progressed into S-phase, and then declined when they progressed into $G_2$ phase and mitosis (Björklund et al., 1990). However, the role that the cell cycle-dependent regulation of both the dCTP pool and the synthesis and degradation of ribonucleotide reductase, particularly the mRNA and the protein of the M2 subunit, might play under hypoxic conditions remains to be elicidated.

Several possible mechanisms involved in the cell cycle arrest during extreme hypoxia remains to be elucidated. To our knowledge no evidence points to possible oxygen-dependent steps and disturbances along the salvage pathway that could block the import and synthesis of pyrimidine deoxynucleotides under extremely hypoxic conditions. On the contrary it has been shown that the dCTP pool is three-quarters restored by the addition of deoxycytidine under hypoxic conditions (Löffler et al., 1983). Therefore, the most likely explanation is that some sort of mechanism is activated or inactivated to prevent cells from the lethal effect of initiating DNA synthesis under conditions of extreme hypoxia. In this context it is interesting to note that both 'normal' cell lines and transformed cell lines such as NHIK 3025, lacking the normal restriction point controls in $G_1$ (i.e. serum starvation) (Rønning & Pettersen, 1984, 1985), respond to oxygen depletion in a similar manner. Thus, cancer cells have not been selected to bypass this highly specific arrest point.

*Cell proliferation under moderately hypoxic conditions*

Under more moderately hypoxic conditions (100–1,300 p.p.m. oxygen), cell cycle arrest in late $G_1$ is less pronounced than under extreme hypoxia, but the rate of DNA synthesis is highly reduced and most cells are unable to complete DNA synthesis under these hypoxic conditions (Figure 4 and 5). However, the presence of deoxycytidine or uridine during moderate hypoxia almost abolishes the inhibition of DNA synthesis but does not establish the normal rate of cell cycle progression. This was previously observed for cells grown in suspension culture, i.e. Ehrlich ascites cells (Löffler, 1987, 1989, 1992; Probst et al., 1989) and indicates that reduced cell proliferation in these and NHIK 3025 cells under conditions of moderate hypoxia (i.e. such as 100 and 1,300 p.p.m. of oxygen) is mainly the result of an insufficient supply of pyrimidine nucleic acid precursors, particularly deoxycytidine (dC can be converted to dTTP in the absence of oxygen). Since cell cycle inhibition was not completely abolished, additional mechanisms regulating the rate of cell proliferation must be present under hypoxic conditions, and there may also be some variation between different cells. In contrast to our results with NHIK 3025 cells, addition of a balanced mixture of deoxycytidine, deoxyadenine and deoxyguanine in 1,300 p.p.m. oxygen was much more efficient than uridine alone in restimulating DNA synthesis in Ehrlich ascites cells (Löffler, 1992). Of particular interest are the data of Figures 4 and 5 showing that the reduced rate of DNA synthesis in the presence of 100 or 1,300 p.p.m. oxygen respectively [where cell respiration is probably not hampered (Froese, 1968; Boag, 1970)] is almost completely counteracted by separate addition of either deoxycytidine or uridine. Both these nucleosides can be used for DNA synthesis through the salvage pathway, while only uridine has to pass through the main route via ribonucleotide reductase (see Figure 1). These results indicate that the reduction of CDP to dCDP by ribonucleotide redustase does not seem to be the limiting step responsible for the reduced dCTP pool observed under moderate hypoxia. Thus, it is more probable that the reduced DNA synthesis observed in NHIK 3025 cells in the presence of 100 or 1,300 p.p.m. oxygen is due to inhibition of the respiratory chain-dependent UMP synthesis at the stage of dihydroorotate dehydrogenase.

Although the dNTP pools are in general sufficient for only a few minutes of DNA synthesis (Reichard, 1988), cells are still able to progress to mid-S-phase (approximately 4 h into normal S-phase) under 100 p.p.m. oxygen. This indicates that the catalytic activity of ribonucleotide reductase in NHIK 3025 cells, although reduced under low levels of oxygen such as 100 p.p.m., is still capable of reducing as much CDP as needed to replicate half the content of DNA in 20 h. As was previously proposed by Probst et al. (1989), the regulation of this enzyme's activity presumably occurs through changing the concentration of the M2-specific tyrosyl radical. This was supported by the observation that *de novo* synthesis of the M2 protein correlates with DNA synthesis and that the half-life of the protein is only 3 h, whereas the level of the M1 protein is constant throughout the cell cycle (Engström et al., 1985). In mammalian ribonucleotide reductase prepared from calf thymus the half-life of the M2 radical was found to be of the order of 10 min under anaerobic conditions (Thelander *et al.*, 1983). Although the continuous presence of oxygen is needed during the catalytic reactions, no specific consumption of oxygen in the catalytic cycle was shown (Thelander *et al.*, 1983). Other groups have shown by use of Electron paramagnetic resonance (EPR) spectroscopy that the intracellular concentration of this radical distinctly decreased under hypoxia and reincreased upon reoxygenation in Ehrlich ascites cells, indicating that oxygen is an essential factor for enzyme activity (Lassmann *et al.*, 1989; Probst *et al.*, 1989).

Both the present and previous results (Probst *et al.*, 1989; Löffler, 1992) point to the size of the dCTP pool and not the size of the purine deoxynucleoside pools as important in regulation of cell proliferation under hypoxic conditions. As shown in Figure 4, addition of deoxyadenine and deoxyguanine during various oxygen concentrations showed no effect on hypoxia-induced inhibition of cell cycle progression. This is in accordance with the earlier observation that the concentration of dATP remains unchanged during hypoxia in Ehrlich ascites cells (Löffler *et al.*, 1983). Furthermore, in the present study, addition of purine deoxynucleosides in combination with deoxycytidine to cells exposed to 100 or 1,300 p.p.m. oxygen had no additive effect to the stimulating effect of deoxycytidine alone. However, in Ehrlich ascites cells the combination of deoxynucleosides was slightly more effective than dC alone (Löffler, 1985*b*). Since degradation of purines is dependent on molecular oxygen (xanthine oxidase oxidises hypoxanthine to xanthine and then to urate) such degradation does not take place under hypoxic conditions and consequently addition of purines has no effect on cell cycle progression during hypoxia (Figure 4c).

### *Cell survival under hypoxic conditions*

We have previously shown that the ability of NHIK 3025 cells to survive exposure to extreme hypoxia is dependent on the position in the cell cycle, with cells in S-phase being most sensitive (Åmellem & Pettersen, 1991). However, cell survival of exponentially growing cells exposed to 100, 1,300 or 5,000 p.p.m. oxygen for 20 h was almost equal to that seen under aerobic conditions (Figure 6) and not influenced by the addition of deoxynucleotides (data not shown). Similar results were obtained with Chinese hamster cells (CHL-F), when 100% of the cells survived 690 p.p.m. oxygen for 28 h (Bedford & Mitchell, 1974). Probst *et al.* (1988) furthermore showed that cell damage and loss of reversibility of the shutdown of replicon initiation occurs in Ehrlich ascites cells at oxygen concentrations below 200 p.p.m.

**Abbreviations:** ribonucleotide reductase (EC 1.17.4.1); dihydroorotate dehydrogenase (EC 1.3.3.1); dNTP, deoxynucleotide triphosphate; dC, deoxycytidine.

The skilful technical assistance of Charlotte Borka, Ursula Prehn Hansen and Wenche Sparre is gratefully acknowledged. The present study was supported by the Norwegian Cancer Society and The Norwegian Radium Hospital Research Foundation.

### References

ÅMELLEM, Ø. & PETTERSEN, E.O. (1991). Cell inactivation and cell cycle inhibition as induced by extreme hypoxia: the possible role of cell cycle arrest as a protection against hypoxia-induced lethal damage. *Cell Prolif.*, **24**, 127–141.

ÅMELLEM, Ø. & PETTERSEN, E.O. (1993). Cell cycle progression in human cells following re-oxygenation after extreme hypoxia: consequences concerning initiation of DNA synthesis. *Cell Prolif.*, **26**, 25–35.

ANDERSON, E.C., PETERSEN, D.F. & TOBEY, R.A. (1967). Biochemical balance and synchronized cell cultures. *Nature*, **215**, 1083–1084.

ANDERSON, G.R. & MATOVCIK, L.M. (1977). Expression of murine sarcoma virus genes in uninfected rat cells subjected to anaerobic stress. *Science*, **197**, 1371–1374.

BEDFORD, J.S. & MITCHELL, J.B. (1974). The effect of hypoxia in the growth and radiation response of mammalian cells in culture. *Br. J. Radiol.*, **47**, 687–696.

BJÖRKLUND, S., SKOG, S., TRIBUKAIT, B. & THELANDER, L. (1990). S-phase-specific expression of mammalian ribonucleotide reductase R1 and R2 subunit mRNAs. *Biochem.*, **29**, 5452–5458.

BJURSELL, K.G. & REICHARD, P. (1973). Effects of thymidine on deoxyribonucleoside triphosphate pools and deoxyribonucleic acid synthesis in Chinese hamster ovary cells. *J. Biol. Chem.*, **248**, 3904–3909.

BOAG, J.W. (1970). Cell respiration as a function of oxygen tension. *Int. J. Radiat. Biol.*, **18**, 475–478.

DEAN, P.N. & JETT, J.H. (1974). Mathematical analysis of DNA distributions derived from flow microfluorometry. *J. Cell Biol.*, **60**, 523–527.

ENGSTRÖM, Y., ERIKSSON, S., JILDEVIK, I., SKOG, S., THELANDER, L. & TRIBUKAIT, B. (1985). Cell cycle-dependent expression of mammalian ribonucleotide reductase. Differential regulation of the two subunits. *J. Biol. Chem.*, **260**, 9114–9116.

FROESE, G. (1962). The respiration of ascites tumour cells at low oxygen concentrations. *Biochim. Biophys. Acta*, **57**, 509–519.

FROESE, G. (1968). The factors affecting tumour oxygenation. *Front. Radiation Ther. Oncol.*, **1**, 16–26.

HEACOCK, C.S. & SUTHERLAND, R.M. (1986). Induction characteristics of oxygen regulated proteins. *Int. J. Radiat. Oncol. Biol. Phys.*, **12**, 1287–1290.

KÆRN, J., TROPÉ, C., KJORSTAD, K.J., ABELER, V. & PETTERSEN, E.O. (1990). Cellular DNA content as a new prognostic tool in patients with borderline tumors of the ovary. *Gyncol. Oncol.*, **38**, 452–457.

KOCH, C.J., KRUUV, J., FREY, H.E. & SNYDER, R.A. (1973*a*). Plateau phase in growth induced by hypoxia. *Int. J. Radiat. Biol.*, **23**, 67–74.

KOCH, C.J., KRUUV, J. & FREY, H.E. (1973*b*). The effect of hypoxia on the generation time of mammalian cells. *Radiat. Res.*, **53**, 43–48.

LASSMANN, G., LIERMANN, B. & LANGEN, P. (1989). Stability and reactivation of tyrosine radicals from ribonucleotide reductase in tumor cells studied by ESR. *Free Radic. Biol. Med.*, **6**, 9.

LINDMO, T. & STEEN, H.B. (1979). Characteristics of a simple high-resolution flow cytometer based on a flow configuration. *Biophys. J.*, **28**, 33–44.

LÖFFLER, M. (1985*a*). Towards a further understanding of the growth-inhibiting action of oxygen deficiency. *Exp. Cell Res.*, **157**, 195–206.

LÖFFLER, M. (1985*b*). Characterization of the deoxynucleoside-dependent reversal of hypoxia-induced inhibition of cell cycle progression in Ehrlich ascites tumor cells. *Eur. J. Cell Biol.*, **39**, 198–204.

LÖFFLER, M. (1987). Restimulation of cell cycle progression by hypoxic tumour cells with deoxynucleosides requires ppm oxygen tension. *Exp. Cell Res.*, **169**, 255–261.

LÖFFLER, M. (1989). The biosynthetic pathway of pyrimidine (deoxy)nucleotides: a sensor of oxygen tension necessary for maintaining cell proliferation? *Exp. Cell Res.*, **182**, 673–680.

LÖFFLER, M. (1992). A cytokinetic approach to determine the range of $O_2$-dependence of pyrimidine(deoxy)nucleotide biosynthesis relevant for cell proliferation. *Cell Prolif.*, **25**, 169–179.

LÖFFLER, M., POSTIUS S. & SCHNEIDER, F. (1978). Anaerobiosis and oxygen recovery: changes in cell cycle distribution of Ehrlich ascites tumor cells grown in vitro. *Virchow's Arch. B Cell Pathol.*, **26**, 359–368.

LÖFFLER, M., SCHIMPFF-WEILAND, G., FOLLMANN, H. (1983). Deoxycytidylate shortage is a cause of $G_1$ arrest of ascites tumor cells under oxygen deficiency. *FEBS Lett.*, **156**, 72–76.

LØVHAUG, D., WIBE, E., OFTEBRO, R., PETTERSEN, E.O. & BRUSTAD, T. (1977). Recovery from X-ray induced damage in human cells grown in culture. *Neoplasma (Bratisl.)*, **24**, 513–520.

MERZ, R. & SCHNEIDER, F. (1983). Growth characteristics of anaerobically treated early and late S-period of Ehrlich ascites tumor cells reaeration. *Z. Naturforsch.*, **38c**, 313–318.

NORDBYE, K. & OFTEBRO, R. (1969). Establishment of four new cell strains from human uterine cervix. *Exp. cell Res.*, **58**, 458.

OFTEBRO, R. & NORDBYE, K. (1969). Establishment of four new cell strains from human uterine cervix. II. *Exp. Cell Res.*, **58**, 459–460.

PETTERSEN, E.O., BAKKE, O., LINDMO, T. & OFTEBRO, R. (1977). Cell cycle characteristics of synchronized and asynchronous populations of human cells and effect of cooling of selected mitotic cells. *Cell Tissue Kinet.*, **10**, 511–522.

PETTERSEN, E.O., JUUL, N.O. & RØNNING, Ø.W. (1986). Regulation of protein metabolism of human cells during and after acute hypoxia. *Cancer Res.*, **46**, 4346–4351.

PETTERSEN, E.O. & LINDMO, T. (1981). Low concentrations of misonidazole counteract effects of extreme hypoxia on cells in S. *Br. J. Cancer*, **43**, 355–366.

PETTERSEN, E.O. & LINDMO, T. (1983). Inhibition of cell-cycle progression by acute treatment with various degrees of hypoxia: modifications induced by low concentrations of misonidazole present during hypoxia. *Br. J. Cancer*, **48**, 809–817.

PETTERSEN, E.O., OFTEBRO, R. & BRUSTAD, T. (1973). X-ray inactivation of human cells in tissue culture under aerobic and extremely hypoxic conditions in the presence and absence of TMPN. *Int. J. Radiat. Biol.*, **24**, 285–296.

PROBST, H. & GEKELER, V. (1988). Oxygen dependent regulation of DNA replication of Ehrlich ascites cells *in vitro* and *in vivo*. In *oxygen Sensing in Tissues*, Acker, H. (ed.). Springer: Berlin.

PROBST, H., SCHIFFER, H., GEKELER, V., KIENZLE-PFEILSTICKER, H., STROPP, U., STÖTZER, K. & FRENZEL-STÖTZER, I. (1988). Oxygen dependent regulation of DNA synthesis and growth of Ehrlich ascites tumor cells *in vitro* and *in vivo*. *Cancer Res.*, **48**, 2053–2060.

PROBST, H., SCHIFFER, H., GEKELER, V. & SCHEFFLER, K. (1989). Oxygen dependent regulation of mammalian ribonucleotide reductase in vivo and possible significance for replicon initiation. *Biochem. Biophys. Res. Commun.*, **163**, 334–340.

PUCK, T.T., CIECIURA, S.J. & FISHER, H.W. (1957). Clonal growth in vitro of human cells with fibroblastic morphology. *J. Exp. Med.*, **106**, 145–165.

REICHARD, P. (1988). Interactions between deoxyribonucleotide and DNA synthesis. *Annu. Rev. Biochem.*, **57**, 349–374.

RØNNING, Ø.W. & PETTERSEN, E.O. (1984). Doubling of cell mass is not necessary in order to achieve cell division in cultured human cells. *Exp. Cell Res.*, **155**, 267–272.

RØNNING, Ø.W. & PETTERSEN, E.O. (1985). Effect of different growth factors on cell cycle traverse and protein growth of human cells in culture. *Exp. Cell Res.*, **157**, 29–40.

RØNNING, Ø.W., LINDMO, T., PETTERSEN, E.O. & SEGLEN, P.O. (1981). Effect of serum step-down on protein metabolism and proliferation kinetics of NHIK 3025 cells. *J. Cell. Physiol.*, **107**, 47–57.

SCIANDRA, J.J., SUBJECK, J.R. & HUGHES, C.S. (1984). Induction of glucose-regulated proteins during anaerobic exposure and of heat-shock proteins after reoxygenation. *Proc. Natl Acad. Sci. USA*, **81**, 4843–4847.

SHI, Y. ÅMELLEM, Ø. & PETTERSEN, E.O. (1993). Proteins specifically regulated under conditions of extreme hypoxia in human cells cultivated in vitro. *APMIS*, **101**, 75–82.

SHRIEVE, D.C. & BEGG, A.C. (1985). Cell cycle kinetics of aerated, hypoxic and re-aerated cells in vitro using flow cytometric determination of cellular DNA and incorporated bromodeoxyuridine. *Cell Tissue Kinet.*, **18**, 641–651.

SHRIEVE, D.C., DEEN, D.F. & HARRIS, J.W. (1983). Effects of extreme hypoxia on the growth and viability of EMT6/SF mouse tumor cells in vitro. *Cancer Res.*, **43**, 3521–3527.

SKOOG, K.L., NORDENSKJÖLD, B.A. & BJURSELL, K.G. (1973). Deoxyribonucleoside-triphosphate pools and DNA synthesis in synchronized hamster cells. *Eur. J. Biochem.*, **33**, 428–432.

SPIRO, I.J., RICE, G.C., DURAND, R.E., STICKLER, R. & LING, C.C. (1984). Cell killing, radiosensitization and cell cycle redistribution induced by chronic hypoxia. *Int. J. Radiat. Oncol. Biol. Phys.*, **10**, 1275–1280.

THELANDER, L., GRÄSLUND, A. & THELANDER, M. (1983). Continual presence of oxygen and iron required from mammalian ribonucleotide reductase: possible regulation mechanisms. *Biochem. Biophys. Res. Commun.*, **110**, 859–865.

VINDELØV, L.L., CHRISTENSEN, I.J. & NISSEN, N.I. (1983*a*). A detergent-trypsin method for the preparation of nuclei for flow cytometric DNA analysis. *Cytometry*, **3**, 323–327.

VINDELØV, L.L., CHRISTENSEN, I.J. & NISSEN, N.I. (1983*b*). Standardization of high-resolution flow cytometric DNA analysis by the simultaneous use of chicken and trout red blood cells as internal reference standards. *Cytometry*, **5**, 328–331.

WALTERS, R.A., TOBEY, R.A. & RATLIFF, R.L. (1973). Cell-cycle-dependent variations of deoxyribonucleoside triphosphate pools in Chinese hamster cells. *Biochim. Biophys. Acta*, **319**, 336–347.